# Immunoprotective Effects of Dietary Fucoidan and Laminarin on Juvenile Blunt Snout Bream (*Megalobrama amblycephala*)

**DOI:** 10.3390/ani16131989

**Published:** 2026-06-27

**Authors:** Ying Wang, Xiaoheng Zhao, Hongping Li, Hujun Cui, Junyan Ma, Ting Zhang, Xu Wang, Xiangning Chen, Hanliang Cheng, Zhujin Ding

**Affiliations:** Jiangsu Key Laboratory of Marine Bioresources and Environment, School of Marine Science and Fisheries, Jiangsu Ocean University, Lianyungang 222005, China; wangying@jou.edu.cn (Y.W.); zhaoxiaoheng@jou.edu.cn (X.Z.); lihongping@jou.edu.cn (H.L.); cuihujun@jou.edu.cn (H.C.); majunyyan@jou.edu.cn (J.M.); zhangting@jou.edu.cn (T.Z.); wangxu@jou.edu.cn (X.W.); xnchen@jou.edu.cn (X.C.); chenghl@jou.edu.cn (H.C.)

**Keywords:** fucoidan, immunoprotective effects, laminarin, *Megalobrama amblycephala*, non-specific immunity

## Abstract

Bacterial diseases occur frequently in freshwater aquaculture in China, highlighting the need for green immune preparations. Fucoidan and laminarin are commonly used feed additives in livestock and poultry industries; however, their application in freshwater aquaculture remains limited. In this study, juvenile *Megalobrama amblycephala* were fed diets supplemented with fucoidan or laminarin, each at 0.5% and 2% (*w*/*w*), for 8 weeks. After the feeding trial, fish were challenged with *Aeromonas hydrophila*. Cumulative mortality rate, tissue bacterial loads, innate immune and antioxidant enzyme activities, and immune-related gene and protein expression were evaluated. Dietary supplementation with 2% fucoidan significantly improved the growth performance and feed utilization of juvenile *M. amblycephala*, while 0.5% fucoidan and 2% laminarin groups only enhanced feed utilization. After *A. hydrophila* infection, cumulative mortality rates for the control, 0.5% and 2% fucoidan, and 0.5% and 2% laminarin groups were 76.67%, 67.78%, 57.78%, 62.22%, and 58.89%, respectively. Both supplements increased post-infection survival, reduced tissue bacterial loads, and enhanced host bactericidal and antioxidative abilities by elevating innate immune and antioxidant enzyme activities. Innate immunity of juvenile *M. amblycephala* was improved by regulation of immune-related gene and protein expression levels. Both additives demonstrated significant immunoprotective effects, markedly reducing mortality and tissue bacterial loads following infection. This study provides a theoretical foundation for developing green immunostimulants and antibiotic alternatives for bacterial diseases in fish.

## Simple Summary

This study addresses the urgent need for green immune preparations to combat bacterial diseases in freshwater aquaculture in China. We explored the potential of two natural feed additives, fucoidan and laminarin, in juvenile *Megalobrama amblycephala*. Over an eight-week period, fish were fed diets supplemented with 0.5% or 2% of these additives. After exposure to *Aeromonas hydrophila*, fish receiving the additives exhibited significantly lower mortality. The additives also decreased bacterial levels in tissues and enhanced the fish’s natural defense capabilities. These results demonstrate that fucoidan and laminarin can effectively safeguard fish from bacterial infections. This research provides a sustainable alternative to antibiotics, contributing to the promotion of healthier aquaculture.

## 1. Introduction

Seaweed polysaccharides are natural polymeric compounds with diverse biological activities that are extracted from marine algae and exhibit a wide variety of types and functions. The names of seaweed polysaccharides are primarily determined by the algae from which they are derived, such as brown, red, and green algal polysaccharides. These substances regulate immunity in humans and farmed animals and enhance antibacterial, antiviral, and antioxidant activities [1]. Thus, seaweed polysaccharides are widely used as food and feed additives, as immune enhancers, and in drug discovery and development [2].

Fucoidan and laminarin are functional polysaccharides derived from brown algae. Fucoidan is primarily sourced from *Laminaria* spp., *Fucus* spp., *Undaria pinnatifida*, *Ascophyllum nodosum*, and *Ecklonia cava* [3], whereas laminarin is mainly derived from *Laminaria* spp., *Ecklonia kurome*, *Eisenia bicyclis*, and *Sargassum henslowianum* [4]. Both polysaccharides have broad potential for application in animal feed and medicine owing to their multiple biological functions, diverse sources, and environmental benefits [5].

Fucoidan is a hydrocolloid composed of fucose and sulfate groups, and its structural diversity is species-dependent. Fucoidan is a sulfated polysaccharide, classified as a fucose-rich glycosaminoglycan, featuring multiple sulfate ester groups (-OSO_3_^−^) localized within the cell wall matrix and intercellular spaces of Phaeophyceae species [6]. Fucoidan enhances immune functions and has anti-coagulation, anti-tumor, antiviral, and antioxidant properties, supporting its broad use in the pharmaceutical and modern food industries [7,8]. Additionally, it alleviates damage to the intestinal physical barrier and mitigates the reduction in the proliferation of intestinal cells caused by inflammatory damage [9]. Therefore, fucoidan is an important multifunctional polysaccharide.

Laminarin is a naturally occurring bioactive polysaccharide and a high-molecular-weight carbohydrate polymer composed of monosaccharide units that are localized in both the intracellular and intercellular compartments of brown algae. Laminarin exhibits diverse biological activities, including hypoglycemic, lipid-regulating, anti-tumor, and antiviral effects, and has the ability to enhance the immunomodulatory capacity of the body [10]. These properties make it a traditional additive in animal feed [11,12,13]. Laminarin enhances specific and non-specific immunity in various aquatic animals and can be used as an additive to improve host immunity [14].

*Megalobrama amblycephala* (blunt snout bream) belongs to the family Cyprinidae and order Cypriniformes. Its broad feeding habits, high survival rate, tender and flavorful meat, and rich nutritional content have made it a popular aquaculture species in China. However, in recent years, due to the deterioration of the aquaculture environment, degradation of germplasm resources, and excessive aquaculture density, the disease resistance of *M. amblycephala* has weakened, leading to frequent disease outbreaks during *M. amblycephala* farming. Bacterial sepsis occurs frequently from July to September. The primary pathogen responsible for this disease is *Aeromonas hydrophila*, which infects *M. amblycephala* and causes extensive hemorrhage on the body surface, base of the fins, and within the abdominal cavity, ultimately resulting in the death of numerous farmed fish. Antibiotics are an effective means of preventing and controlling bacterial diseases in aquaculture. However, their overuse leads to bacterial resistance and raises concerns about the safety of aquatic products. In the context of current antibiotic reduction and substitution strategies, an increasing number of scholars are focusing on alternatives to antibiotics [15].

Identifying green and effective antibiotic substitutes is therefore crucial for healthy *M. amblycephala* production. Fucoidan and laminarin have been widely used as immunostimulants in the livestock and poultry industries. Our previous study indicates that fucoidan and laminarin can enhance the phagocytosis of *M. amblycephala* macrophages, improving the host’s bacterial clearance capacity [16]. At the molecular level, tumor necrosis factor alpha (TNFα) and interleukin-1 beta (IL1β) are important pro-inflammatory cytokines mediating the early antibacterial immune response in fish [17,18], while inducible nitric oxide synthase (iNOS) also serves as a critical inflammation-associated enzyme in inflammatory regulation [19]. Evaluating the expression of these molecules can help elucidate how dietary immunostimulants modulate host inflammatory responses following pathogen challenge.

Therefore, the present study investigated the effects of fucoidan and laminarin as immunostimulants on the cumulative mortality rate, tissue bacterial load, pathological injury, and non-specific immunity in juvenile *M. amblycephala*. The objective of this study was to ascertain the immunoprotective effects of fucoidan and laminarin in *M. amblycephala*, thereby establishing a foundation for preventing and treating bacterial diseases in *M. amblycephala*.

## 2. Materials and Methods

### 2.1. Ethics Statement

This study was approved by the Ethics Committee for Laboratory Animals of Jiangsu Ocean University (Protocol No.: 2020-37; Approval Date: 1 September 2019). All experimental procedures involving animals were conducted strictly in accordance with the Declaration of Helsinki and the Regulations on the Administration of Laboratory Animals in China.

### 2.2. Dietary Formulation

According to the industry standard for compound feed for *M. amblycephala* (SC/T 1074-2022) [20], a basic isonitrogenous and isoenergetic basal diet was prepared using fish meal, soybean meal, cottonseed meal, and rapeseed meal as protein sources; soybean oil as a fat source; and wheat middlings as a carbohydrate source. The experimental diets of the fucoidan and laminarin groups were formulated by adding 0.5% and 2% (*w*/*w*) seaweed polysaccharides to the basal diet (Table 1). The experimental diets were formulated to be isonitrogenous and isoenergetic, with crude protein and gross energy levels showing only minor analytical variation across groups. Fucoidan and laminarin are commercial polysaccharides that have been utilized in previous studies [16]. Fucoidan was purchased from Macklin Inc. (Shanghai, China), sourced from brown algae, with a purity of 98% and molecular weight of 58.5 kDa, and mainly consists of the monosaccharides L-fucose and galactose. Laminarin was obtained from Xagelinbio Co., Ltd. (Xi’an, China) and derived from *Laminaria japonica*, with a purity of 98% and molecular weight of 230.2 kDa, and its main constituent monosaccharide is glucose.

All powdered ingredients were weighed and mixed for 10 min, after which distilled water was added and mixed for an additional 15 min. A pelletizing aperture of approximately 1.5 mm was set according to the size of the experimental fish. The pellets were crushed into granules and dried in a drying oven to ensure a water content < 10%.

### 2.3. Fish Rearing and Growth Performance

**Figure 1:**
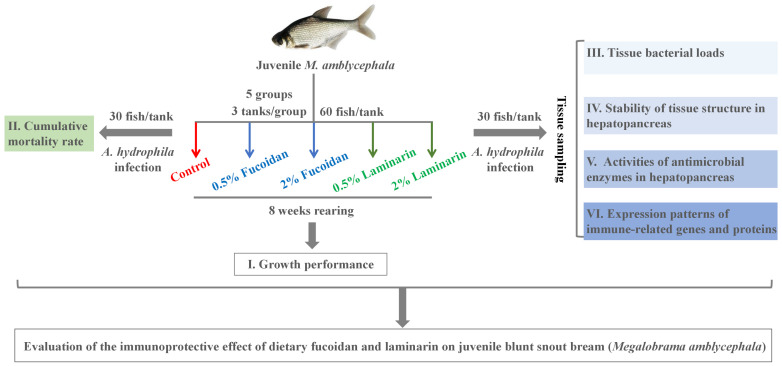
Flowchart of the experimental design in the present study. The experiments included fish rearing, bacterial challenge, sample collection, and immune index analysis.

Juvenile *M. amblycephala* were obtained from a commercial fish farm in Huzhou, China. Prior to the formal feeding trial, the fish were reared on a self-formulated basal diet (free of fucoidan or laminarin) during a 2-week acclimation period, which allowed full recovery from transport stress and stable adaptation to laboratory culture conditions. After acclimation, all fish underwent visual health screening, and only individuals with normal swimming and feeding behavior, as well as no visible surface damage or external lesions, were selected for the experiment. The formulation and proximate nutrient composition of the basal diet are presented in Table 1. Experimental diets were prepared by adding graded levels of fucoidan or laminarin to the basal formula via equal replacement of wheat middlings, with all other dietary ingredients kept constant. The experimental design and procedure for fish rearing, bacterial challenge, and immune evaluation are detailed in Figure 1. In total, 900 experimental fish with a body weight of 9.96 ± 1.48 g were randomly assigned to 5 groups: control, 0.5% fucoidan, 2% fucoidan, 0.5% laminarin, and 2% laminarin groups. Each group consisted of three replicate 240 L capacity tanks, with 60 fish per tank.

The experimental fish were maintained in an indoor freshwater recirculating system consisting of 15 tanks, all equipped with identical aeration and water flow at a rate of 1 L/min. The experimental fish were reared for 8 weeks and fed to apparent satiation four times daily (at 08:00, 11:00, 14:00, and 17:00). The daily feeding ratio under this satiation regime was approximately 3% of body weight. Water was renewed daily to maintain acceptable water quality. The water temperature was maintained at 28 ± 1 °C; the pH was approximately 7.2; ammonia, nitrogen, and nitrite levels were <0.1 mg/L; and the dissolved oxygen concentration was >6.0 mg/L. The initial and final body weights, as well as the total feed intake, were measured before and after the rearing experiment. The corresponding formulae for calculating the growth index were as follows:

Weight gain rate (%) = [(final body weight − initial body weight)/initial body weight] × 100(1)

Feed conversion ratio = total feed intake/weight gain(2)

### 2.4. Bacterial Challenge and Sample Collection

The bacterial challenge was conducted after the 8-week rearing experiment, as previously described [15]. Fish from the control, 0.5% fucoidan, 2% fucoidan, 0.5% laminarin, and 2% laminarin groups (60 fish per tank) were divided into 2 categories: 30 fish per tank were used for mortality recording, and 30 fish per tank were used for post-challenge sampling, with the tank volume adjusted to 90 L.

The experimental fish, with a body weight of 35.48 ± 7.20 g, were injected intraperitoneally with 0.1 mL (1 × 10^8^ colony-forming units/mL) of *A. hydrophila* (lethal dose 50%). After being anesthetized with 3-aminobenzoic acid ethyl ester methane sulfonate (MS-222; Merck KGaA, Darmstadt, Germany), nine fish from each group (i.e., three fish per tank, three replicate tanks per group) were randomly dissected at 0, 6, 24, and 72 h post-infection (hpi), and the hepatopancreas, kidneys, intestines, and gills were collected. The hepatopancreas was used to examine the bacterial load, observe histopathological structures, detect enzyme activities, and analyze the expression of target genes and proteins. The kidneys were collected to analyze the tissue bacterial load and expression of the target genes and proteins. The intestines and gills were sampled to detect the tissue bacterial load.

### 2.5. Histological Assay

Hematoxylin and eosin (H&E) staining of tissue sections was conducted to detect histological structures of *M. amblycephala* hepatopancreas. The tissue samples were soaked in a 4% paraformaldehyde solution for 24 h at 4 °C. The samples were then dehydrated using an ethanol gradient, cleaned with xylene, embedded in paraffin blocks, and sectioned at a 4 µm thickness on a microtome (Leica, Wetzlar, Germany). Samples were then immersed in a 40 °C water bath, affixed to glass slides, and dried in an oven at 40 °C overnight. After removing the paraffin and replenishing it with water, the sections were stained with H&E. A light microscope was used for observation and photography (Olympus, Tokyo, Japan).

### 2.6. Innate Immune and Antioxidant Enzyme Activities

The excised hepatopancreases were weighed and homogenized at a tissue weight (g): volume of phosphate-buffered saline (mL) ratio of 1:9, using a high-throughput macerator. Following centrifugation at 2500 rpm for 10 min, the supernatant was extracted to assess the activities of hepatopancreatic innate immune and antioxidant enzymes. The activities of acid phosphatase (ACP), alkaline phosphatase (AKP), catalase (CAT), superoxide dismutase (SOD), glutathione S-transferase (GST), and lysozyme (LZM) were determined using the corresponding enzyme activity detection kits (Nanjing Jiancheng Bioengineering Institute, Nanjing, China), according to the manufacturer’s instructions.

### 2.7. Total RNA Isolation and cDNA Preparation

Total RNA was isolated from the hepatopancreas and kidney samples using an RNA Easy Fast Tissue Kit (TIANGEN, Beijing, China), according to the manufacturer’s instructions. The quality and concentration of total RNA were determined using agarose gel electrophoresis and a NanoDrop 2000 (Thermo Fisher Scientific, Wilmington, DE, USA), respectively. The cDNA was synthesized using a PrimeScript^®^ RT reagent kit with gDNA Eraser (TaKaRa, Dalian, China), following the manufacturer’s protocol, and stored at −20 °C for use in the real-time quantitative reverse transcription polymerase chain reaction (qRT-PCR) assay.

### 2.8. Real-Time qRT-PCR

The expression patterns of tight junctions and immune-related genes were analyzed using qRT-PCR, as previously reported [16]. Briefly, qRT-PCR was performed using an ABI StepOnePlus Real-Time PCR System (PerkinElmer Applied Biosystems, Waltham, MA, USA) with a QuantiNova™ SYBR^®^ Green PCR Kit (TaKaRa), according to the manufacturer’s protocol. The reaction system, with a total volume of 20 µL, comprised 10.0 µL of 2× SYBR Green PCR Master Mix, 2.0 µL of QN ROX Reference Dye, 0.6 µL of forward primer, 0.6 µL of reverse primer, 1.0 µL of cDNA, and 5.8 µL of RNase-free water. The reaction process was as follows: pre-denaturation at 95 °C for 2 min, followed by 40 cycles of denaturation at 95 °C for 5 s, and one-step annealing and elongation at 60 °C for 10 s. Melting curve analysis was then performed at 95 °C for 15 s and 30 °C for 1 min to assess the specificity of the qRT-PCR.

The relative expression levels of the target genes were measured using the 2^−∆∆Ct^ method, in terms of the threshold cycle (Ct) value [21]; *glyceraldehyde-3-phosphate dehydrogenase* (*GAPDH*) was selected as the internal reference gene based on an analysis conducted using geNorm software (Version 3.4) [22]. All reactions were performed in triplicate, and the primers used are listed in Table 2. The gene expression levels in the control group were set to 1, and the relative expression levels of the experimental groups are presented as fold-changes.

*A. hydrophila* abundance in various tissues of *M. amblycephala* from the different groups was monitored by measuring the levels of *A. hydrophila 16S rRNA* transcripts, which were calculated based on the Ct values obtained from qRT-PCR.

### 2.9. Western Blotting

Western blotting was performed as previously described [23]. Proteins were separated on a 4–20% sodium dodecyl sulfate–polyacrylamide gel electrophoresis gel and transferred onto polyvinylidene difluoride (PVDF) membranes using a Trans-Blot apparatus (BioRad, Hercules, CA, USA) for 2 h at 80 V. Non-specific reactivity was blocked with 5% (*w*/*v*) skim milk powder in tris-buffered saline (150 mM NaCl, 20 mM Tris-base, pH 7.4) for 1 h at 30 °C. The PVDF membranes were incubated with primary antibodies at the appropriate concentration overnight at 4 °C (Table 3), then washed thrice, and incubated with horseradish peroxidase (HRP)-conjugated goat anti-rabbit IgG (Beyotime, Shanghai, China; 1:2000 dilution) for 1 h at 30 °C. Finally, a DAB HRP Color Development Kit (Beyotime) was used to detect the immunoreactive bands.

### 2.10. Statistical Analysis

Data are presented as mean values ± standard error (SE). Statistical significance was assessed using one-way analysis of variance, and multiple comparisons were performed using Tukey’s method in SPSS 25.0. Statistical significance was set at *p* < 0.05.

## 3. Results

### 3.1. Effects of Dietary Fucoidan and Laminarin on Growth Performance and Feed Utilization in Juvenile M. amblycephala

The effects of dietary supplementation with fucoidan and laminarin on the growth performance and feed utilization of juvenile *M. amblycephala* are shown in Table 4. Compared with the control group, the weight gain rate was significantly higher only in the 2% fucoidan group (*p* < 0.05), while no significant difference was observed in the other polysaccharide-supplemented groups. The feed conversion ratio was significantly reduced in the 0.5% fucoidan, 2% fucoidan, and 2% laminarin groups compared with the control group (*p* < 0.05), while no significant reduction was observed in the 0.5% laminarin group. These results indicated that dietary supplementation with 2% fucoidan could effectively improve the growth performance and feed utilization of juvenile *M. amblycephala*, while laminarin only exerted beneficial effects on feed utilization at the 2% supplementation level.

### 3.2. Dietary Supplementation with Fucoidan and Laminarin Improved the Survival Rate of Juvenile M. amblycephala Following Infection

**Figure 2:**
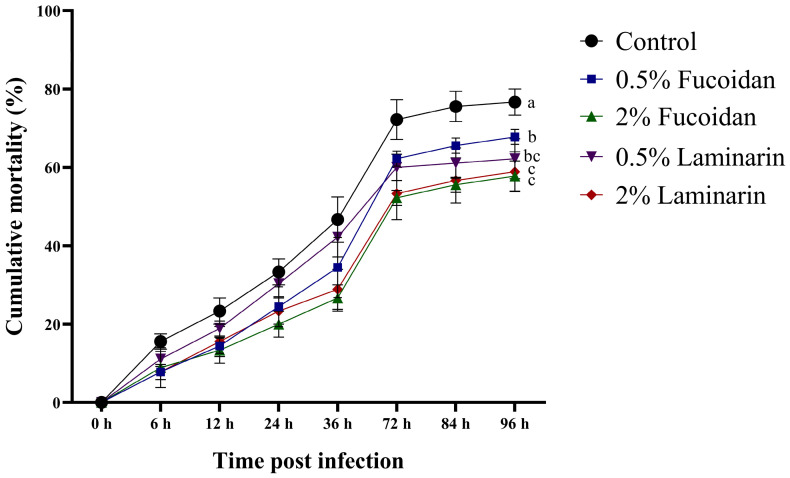
Effect of dietary fucoidan and laminarin on mortality of juvenile *M. amblycephala* post-*A. hydrophila* infection. Different letters indicate statistically significant differences among different groups (*p* < 0.05).

The cumulative mortality rates of *M. amblycephala* in all groups increased markedly after *A. hydrophila* infection. A rapid and significant increase was observed from 6 hpi to 72 hpi, followed by a slow rise thereafter. Numerically, the highest cumulative mortality rate in all groups was recorded at 96 hpi, with no statistically significant difference observed between 84 hpi and 96 hpi within each group (Figure 2). At all time points, cumulative mortality was higher in the control group than in the fucoidan- and laminarin-supplemented groups. The final cumulative mortality rates in the control, 0.5% and 2% fucoidan, and 0.5% and 2% laminarin groups were 76.67%, 67.78%, 57.78%, 62.22%, and 58.89%, respectively. The groups supplemented with 2% polysaccharides demonstrated significantly stronger immunoprotective effects than the control and 0.5% polysaccharide-supplemented groups (*p* < 0.05). The results indicated that dietary supplementation with fucoidan and laminarin, particularly in the high-dose groups, conferred immunoprotective effects on juvenile *M. amblycephala*.

### 3.3. Effect of Dietary Supplementation with Fucoidan and Laminarin on Tissue Bacterial Loads of Juvenile M. amblycephala

**Figure 3:**
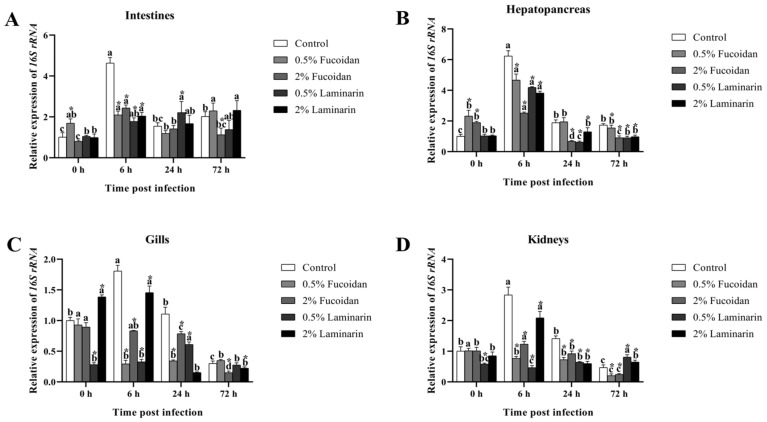
Bacterial loads in the tissues of juvenile *M. amblycephala* detected using qRT-PCR based on the transcript levels of *A. hydrophila 16S rRNA*. Bacterial loads in the: (**A**) intestines, (**B**) hepatopancreas, (**C**) gills, and (**D**) kidneys. Asterisks denote statistically significant differences among groups at a particular time point (*p* < 0.05), and different letters indicate statistically significant differences for a certain group across various time points (*p* < 0.05).

The transcript levels of *A. hydrophila 16S rRNA* in juvenile *M. amblycephala* were measured using qRT-PCR to compare bacterial loads across different groups at various time points and in different tissues. As shown in Figure 3, the bacterial load increased markedly at 6 hpi but was significantly higher in the control group than in the polysaccharide-supplemented groups (*p* < 0.05). Subsequently, the bacterial load gradually decreased and was consistently lower in most polysaccharide-supplemented groups than in the control group. Fucoidan and laminarin supplementation substantially decreased the bacterial load in the tissues, thereby protecting the host from infection.

### 3.4. Dietary Supplementation with Fucoidan and Laminarin Maintained the Stability of the Hepatopancreas Tissue Structures in Juvenile M. amblycephala

**Figure 4:**
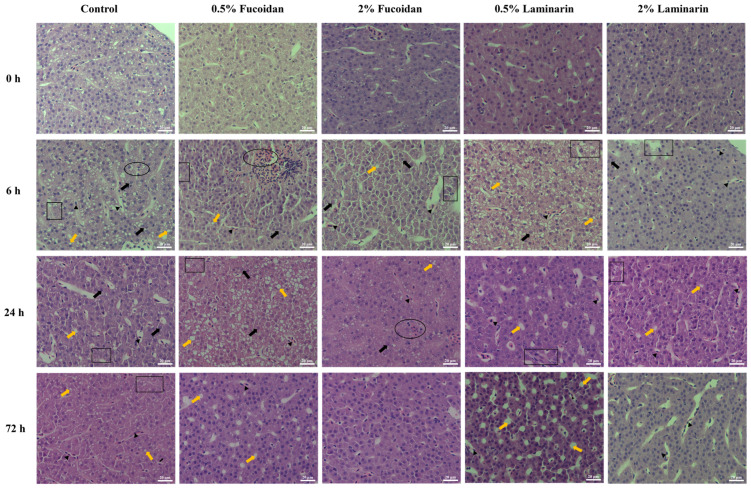
Histopathological changes in the hepatopancreas of juvenile *M. amblycephala* before and after infection with *A. hydrophila*. Black arrow, hydropic degeneration; yellow arrow, steatosis; rectangle, hepatocyte necrosis; triangle, sinusoidal congestion; oval, hemorrhage. Scale bars = 20 µm.

Pathological changes in the hepatopancreas of juvenile *M. amblycephala* were analyzed using H&E staining (Figure 4). Prior to *A. hydrophila* infection (0 hpi), the hepatopancreatic cells of all groups were neatly arranged, exhibiting a round or oval shape. They had intact cellular structures with distinct boundaries, and no marked pathological changes were observed.

At 6 hpi, the hepatopancreas of the control group exhibited marked pathological changes. The hepatopancreatic blood sinusoids were congested, and the cells were edematous, degenerate, and fatty. The cytoplasm contained numerous round lipid vacuoles of varying sizes, and the nuclei were compressed to one side. Some hepatocytes were enlarged with pale cytoplasm. In the most severe areas of the lesions, the hepatocytes were ruptured and necrotic, and their nuclei were lysed. In the hepatopancreas of fish in the 0.5% fucoidan group, hepatocellular steatosis and edematous degeneration were also observed, with congestion of the hepatopancreatic sinusoids, focal necrosis, and hemorrhaging in some tissues. In the other groups, extensive hepatocellular steatosis, partial edematous metaplasia, congestion of hepatopancreatic blood sinusoids, and hepatocellular necrosis in the hepatopancreas were observed.

At 24 hpi, the hepatopancreas in the control group exhibited severe tissue damage, including hepatocellular necrosis, extensive steatosis, and edematous degeneration. In the 0.5% fucoidan and 0.5% laminarin groups, numerous hepatopancreatic sinusoids were congested, and a minor fraction of hepatocytes were necrotic, whereas the hepatopancreas of the 2% fucoidan and 2% laminarin groups showed only a few necrotic hepatocytes. At 72 hpi, hepatocytes exhibiting steatosis remained in the hepatopancreas of fish in both the control and 0.5% fucoidan groups, whereas fish in the other groups did not exhibit any apparent pathological features.

Histopathological analyses indicated that dietary fucoidan and laminarin reduced tissue damage caused by *A. hydrophila* infection in the hepatopancreas of juvenile *M. amblycephala*, thereby maintaining the stability of tissue structures, particularly in the 2% fucoidan and 2% laminarin groups.

### 3.5. Dietary Supplementation with Fucoidan and Laminarin Enhanced Hepatopancreatic Innate Immune and Antioxidant Enzyme Activities in Juvenile M. amblycephala

**Figure 5:**
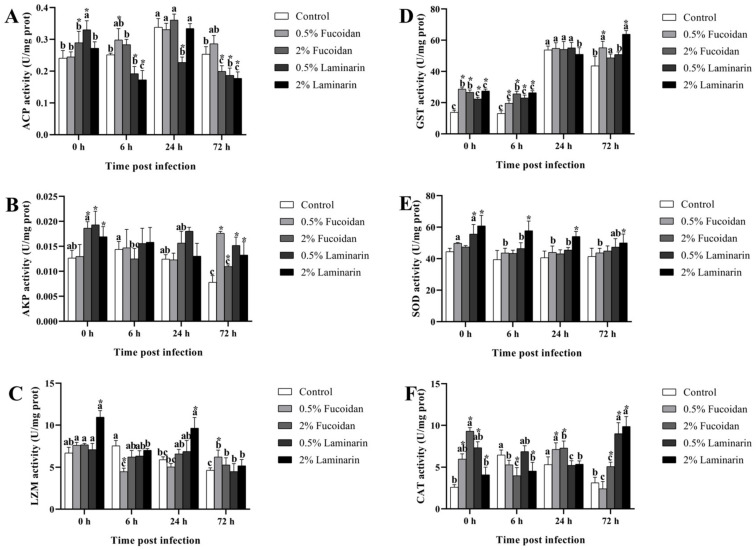
Effects of fucoidan and laminarin supplementation on the activities of hepatopancreatic innate immune and antioxidant enzymes in juvenile *M. amblycephala*. Activities of: (**A**) acid phosphatase (ACP), (**B**) alkaline phosphatase (AKP), (**C**) lysozyme (LZM), (**D**) glutathione S-transferase (GST), (**E**) superoxide dismutase (SOD), and (**F**) catalase (CAT). Asterisks indicate statistically significant differences among different groups at a specific time point (*p* < 0.05), and different letters indicate statistically significant differences within a certain group across various time points (*p* < 0.05).

To investigate the effects of dietary fucoidan and laminarin on antimicrobial and antioxidant capacity, the activities of innate immune and antioxidant enzymes in the hepatopancreas of juvenile *M. amblycephala* were examined. ACP activity did not differ markedly between the fucoidan-supplemented groups and the control group at any time point, with a pattern of upregulation followed by downregulation post-infection (Figure 5). However, the ACP activity in the laminarin-supplemented groups exhibited a different trend compared to that in the other groups, possessing higher activities before infection and maintaining relatively lower activities at most time points post-infection (*p* < 0.05). Thus, dietary fucoidan and laminarin exhibited distinct regulatory effects on host ACP activity.

AKP activity in some fucoidan- or laminarin-supplemented groups was significantly higher than that in the control group, both before and after infection with *A. hydrophila* (*p* < 0.05). This suggested that fucoidan and laminarin supplementation improved the antimicrobial capacity of the host.

LZM activity remained relatively stable across groups and at most time points. The 2% laminarin group demonstrated significantly higher LZM activity than the other groups at both 0 hpi and 24 hpi (*p* < 0.05), and the 0.5% fucoidan group exhibited the lowest activity at 6 hpi (*p* < 0.05), indicating that dietary fucoidan and laminarin resulted in distinct regulation of the antimicrobial capacity of the host.

Following infection, GST activity gradually increased in all groups, with the fucoidan- and laminarin-supplemented groups exhibiting higher GST activity than the control group at most time points (*p* < 0.05). The SOD activity remained relatively stable before and after infection but was consistently higher in the 2% laminarin group than in the other groups (*p* < 0.05).

Before infection, CAT activity was generally higher in all polysaccharide-supplemented groups than in the control group (*p* < 0.05). After infection, CAT activity in the control group initially increased and then decreased, whereas CAT activity in the supplemented groups initially decreased and then increased. The supplemented groups generally had higher CAT activity than the control group, except at 6 hpi.

Dietary fucoidan and laminarin improved the antibacterial ability of juvenile *M. amblycephala* by modulating the activities of ACP, AKP, and LZM and enhanced the antioxidant ability by influencing the activities of CAT, SOD, and GST. Moreover, the regulatory effects of fucoidan and laminarin on these enzyme activities varied in a dose- and time-dependent manner.

### 3.6. Effect of Dietary Addition of Fucoidan and Laminarin on Expression of Immune-Related Genes in Juvenile M. amblycephala

**Figure 6:**
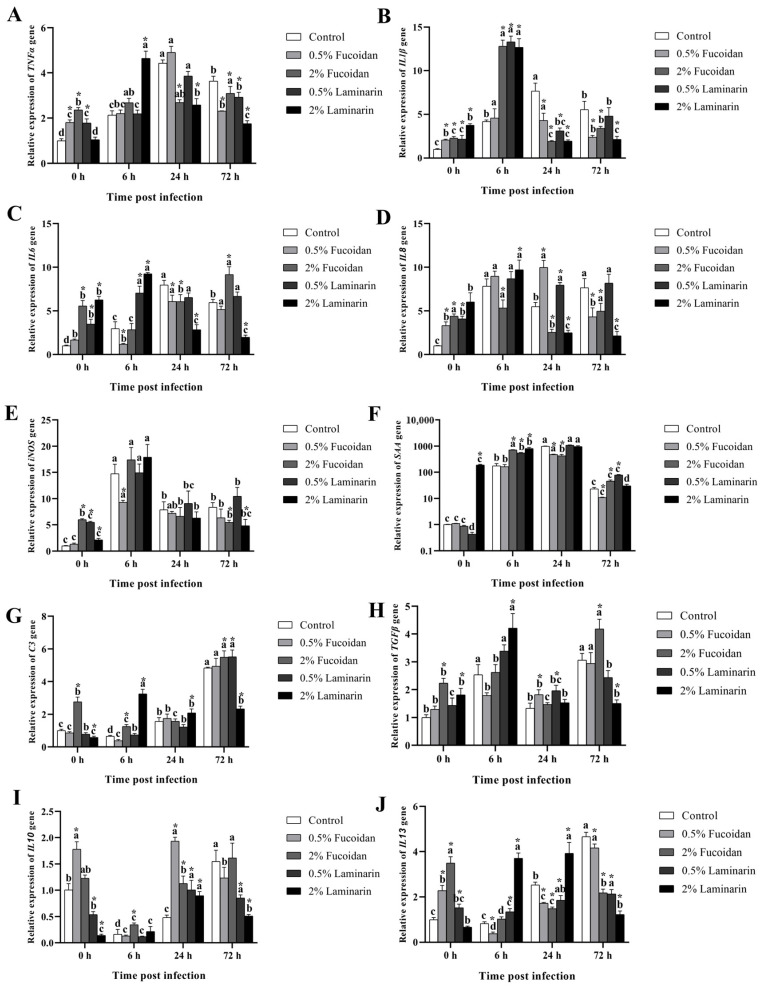
Expression patterns of immune-related genes in the hepatopancreas of juvenile *M. amblycephala* upon infection. Detected genes included: (**A**) *tumor necrosis factor alpha* (*TNFα*), (**B**) *interleukin-1 beta* (*IL1β)*, (**C**) *interleukin-6* (*IL6)*, (**D**) *interleukin-8* (*IL8*), (**E**) *inducible nitric oxide synthase* (*iNOS*), (**F**) *serum amyloid A* (*SAA*), (**G**) *complement component 3* (*C3*), (**H**) *transforming growth factor beta* (*TGFβ*), (**I**) *interleukin-10* (*IL10*), and (**J**) *interleukin-13* (*IL13*). *Glyceraldehyde-3-phosphate dehydrogenase* (*GAPDH*) was selected as the reference gene. Data are mean values ± SE. Asterisks indicate statistically significant differences among different groups at a specific time point (*p* < 0.05), and different letters indicate statistically significant differences within a certain group across various time points (*p* < 0.05).

The expression of immune-related genes in the hepatopancreas and kidneys of juvenile *M. amblycephala* was examined using qRT-PCR. As shown in Figure 6, the pro-inflammatory cytokines *TNFα*, *IL1β*, *interleukin-6* (*IL6*), and *interleukin-8* (*IL8*), as well as the inflammation-associated enzyme *iNOS* and the major acute-phase protein *serum amyloid A* (*SAA*), exhibited similar expression patterns in the hepatopancreas, with increased expression levels at 6 hpi in all groups, which gradually decreased thereafter. Expression in most of the polysaccharide-supplemented groups was higher than that in the control group before 6 hpi, particularly in the 2% laminarin group (*p* < 0.05). However, expression in most polysaccharide-supplemented groups was lower after 24 hpi, which may have prevented body damage from an excessive inflammatory response.

Upon infection, the anti-inflammatory cytokines *interleukin-10* (*IL10*), *interleukin-13* (*IL13*) and *transforming growth factor beta* (*TGFβ*), as well as *complement component 3* (*C3*), exhibited similar expression patterns, with the levels decreasing or remaining relatively low at 6 hpi and 24 hpi, followed by subsequent upregulation at 72 hpi. The expression levels in the 2% laminarin group were initially higher than those in the control group but were substantially lower at the later stages to maintain the immune balance of the host.

**Figure 7:**
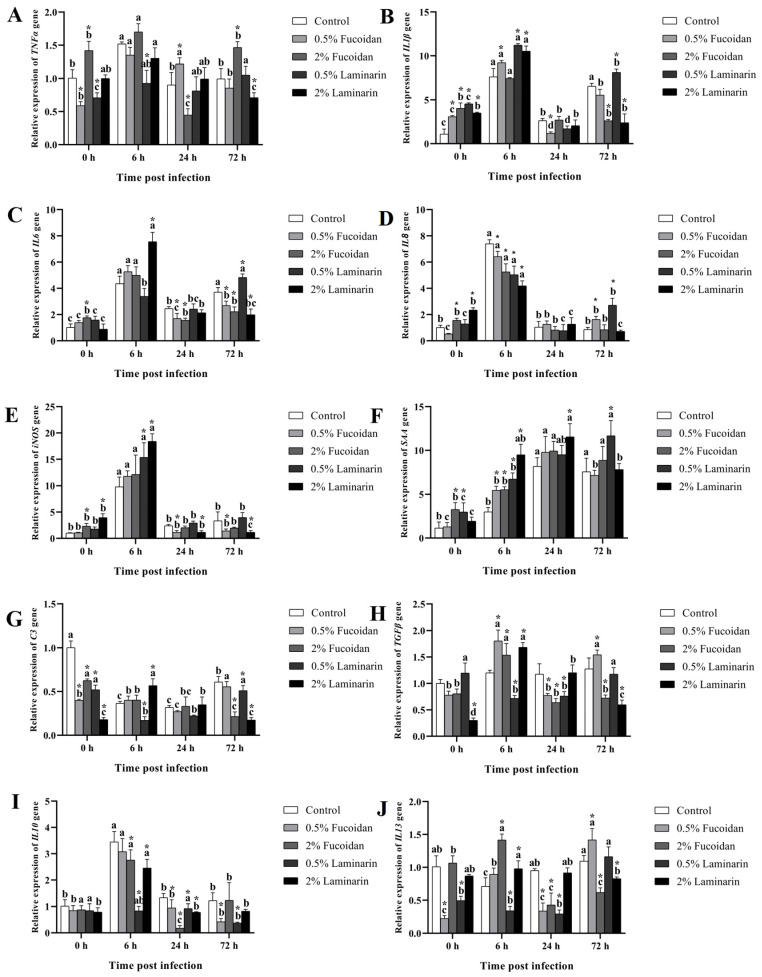
Expression patterns of immune-related genes in the kidneys of juvenile *M. amblycephala* upon infection. Detected genes included: (**A**) *tumor necrosis factor alpha* (*TNFα*), (**B**) *interleukin-1 beta* (*IL1β)*, (**C**) *interleukin-6* (*IL6)*, (**D**) *interleukin-8* (*IL8*), (**E**) *inducible nitric oxide synthase* (*iNOS*), (**F**) *serum amyloid A* (*SAA*), (**G**) *complement component 3* (*C3*), (**H**) *transforming growth factor beta* (*TGFβ*), (**I**) *interleukin-10* (*IL10*), and (**J**) *interleukin-13* (*IL13*). *Glyceraldehyde-3-phosphate dehydrogenase* (*GAPDH*) was selected as the reference gene. Data are mean values ± SE. Asterisks indicate statistically significant differences among different groups at a specific time point (*p* < 0.05), and different letters indicate statistically significant differences within a certain group across various time points (*p* < 0.05).

Expression patterns of *IL1β*, *IL6*, *IL8*, *iNOS* and *SAA* in the kidneys were similar among the different groups, with an increase from 6 hpi, followed by decreased expression (Figure 7). Expression levels in some polysaccharide-supplemented groups were generally higher than those in the control group before infection and at 6 hpi, whereas most polysaccharide-supplemented groups exhibited lower expression levels at 24 and 72 hpi, which may have been a response to prevent over-immunity. Unlike other pro-inflammatory cytokines, *TNFα* expression displayed significant temporal and intergroup fluctuations post-infection: 2% fucoidan induced consistent upregulation at 0, 6, and 72 hpi but marked suppression at 24 hpi, while different doses of fucoidan and laminarin exerted distinct regulatory effects relative to the control.

*TGFβ*, *IL13*, and *C3* exhibited similar expression patterns in the kidneys upon infection. Expression levels in all groups fluctuated upon infection, with those in some polysaccharide-supplemented groups being lower than those in the control group post-infection. However, *IL10* expression patterns differed from those of the other anti-inflammatory cytokines, with expression levels increasing substantially at 6 hpi. This may have prevented early over-immunity and ensured an immune balance between pro-inflammatory and anti-inflammatory responses.

### 3.7. Expression Patterns of Immune-Related Proteins in Juvenile M. amblycephala Regulated by Dietary Fucoidan and Laminarin

The effects of fucoidan and laminarin supplementation on the expression of immune-related proteins were examined using Western blotting (Supplemental Figures S2–S4). Collectively, the expression profiles of the pro-inflammatory proteins TNFα, IL1β, and iNOS differed significantly between the hepatopancreas and kidneys following *A. hydrophila* challenge, exhibiting distinct tissue-specific temporal dynamics and dose-dependent regulation by fucoidan and laminarin across treatment groups.

In the hepatopancreas (Supplemental Figure S2B), TNFα and IL1β were predominantly downregulated in most polysaccharide-treated groups compared with the control group at 6, 24 and 72 hpi. In the kidneys (Supplemental Figure S2C), TNFα exhibited a distinct time-dependent biphasic response: suppressed in most treatment groups at 6 and 24 hpi, but significantly upregulated in most treatment groups at 72 hpi; in contrast, IL1β was consistently upregulated in most polysaccharide-treated groups at all post-infection time points, with especially pronounced upregulation observed at 72 hpi.

The iNOS protein showed distinct tissue-specific temporal patterns. In the hepatopancreas (Supplemental Figure S2B), iNOS gradually increased post-infection in all groups; fucoidan and laminarin significantly suppressed iNOS at most time points, except at 72 hpi in the 2% fucoidan group, in which it was significantly higher than in the control. In the kidneys (Supplemental Figure S2C), iNOS decreased at 6 hpi and then rose gradually in the control group, while all treatment groups maintained significantly lower levels than controls at all time points. Overall, dietary fucoidan and laminarin modulate key inflammatory mediators in a tissue- and time-dependent manner in *M. amblycephala* post-bacterial infection.

## 4. Discussion

### 4.1. Effects of Dietary Addition of Fucoidan and Laminarin on Growth Performance and Feed Utilization

Supplementing diets with appropriate amounts of fucoidan and laminarin can improve feed utilization rates and promote the growth of farmed animals. Dietary supplementation with fucoidan can slightly enhance the growth of juvenile *Larimichthys crocea* by accelerating digestive maturation, regulating the intestinal flora spectrum, and substantially improving the final body weight, weight gain rate, and specific growth rate of juvenile *L. crocea* [24]. Similarly, dietary laminarin can substantially promote fish growth by increasing the body weight gain rate, specific growth rate, and feed conversion rate of *L. crocea* [25]. Moreover, dietary fucoidan supplementation can also effectively enhance the growth performance of *Oreochromis niloticus* [26] and *Lutjanus argentimaculatus* [27], and dietary laminarin supplementation effectively improves the growth performance of *Epinephelus coioides* [28], *Micropterus salmoides* [13], and *Ictalurus punctatus* [29]. Most aquaculture studies apply 0.1–2.0% dietary fucoidan or laminarin; we selected 0.5% and 2% as typical low and high doses within this effective range to examine dose-dependent effects. Excessive polysaccharides may reduce feed palatability and impose digestive stress; since doses above 2% were not evaluated, the adverse-effect threshold for *M. amblycephala* is unconfirmed.

The present study found that dietary supplementation with 2% fucoidan significantly increased the weight gain rate of juvenile *M. amblycephala*, and most polysaccharide-supplemented groups decreased the feed conversion ratio. Only the 0.5% laminarin group showed no significant improvement in FCR, indicating that this dosage was below the effective threshold for regulating digestive function. Notably, both groups receiving 2% supplementation achieved the lowest FCR values across all treatments, demonstrating that high doses of fucoidan and laminarin are equally effective at improving feed utilization.

The differential growth-promoting effects of fucoidan and laminarin may be attributed to their distinct structural properties and biological activities, while the underlying mechanisms involving digestive enzyme activity, gut microbiota composition and nutrient metabolism remain to be further elucidated. Since no supplementation levels above 2% were tested in this study, we cannot confirm whether 2% is the physiological peak for growth promotion or assess potential adverse effects of higher doses. Moreover, growth effects are also influenced by trial duration and fish developmental stage, so further systematic studies are required.

### 4.2. Dietary Addition of Fucoidan and Laminarin Reduces the Mortality Rate Following Bacterial Infection

The effects of dietary fucoidan and laminarin supplementation on infection in aquatic animals have been previously reported. Fucoidan addition to the diet decreases cumulative mortality and mitigates damage from cadmium chloride immunosuppression in *Cystoseira trinodis* [30] and improves the survival rate and disease resistance of *C. trinodis* against white spot syndrome virus [31]. Dietary laminarin is reported to protect *L. crocea* from *Pseudomonas plecoglossicida* infection [25]. Cumulative mortality rates in the fucoidan- and laminarin-supplemented groups in the present study were substantially lower than those in the control group post-infection, suggesting that dietary fucoidan and laminarin provided substantial immune protection to juvenile *M. amblycephala*. These results indicated that fucoidan and laminarin addition to the diet can enhance the infection resistance in these fish.

### 4.3. Dietary Fucoidan and Laminarin Affected the Bacterial Load in Tissues

The bacterial load is an important indicator of the level of pathogenic infection. Immune agents, including immunostimulants and vaccines, can inhibit pathogen infection by reducing the tissue bacterial load. In fucoidan-supplemented groups, the *Vibrio parahaemolyticus* load in *Penaeus monodon* tissues decreased from the 10th to the 21st day of challenge, indicating that fucoidan enhanced bacterial clearance from the host [32]. Supplementation with 3 g/kg seaweed polysaccharides reduces the abundance of pathogenic microbes in *Litopenaeus vannamei* [33]. When used as an adjuvant, inositol substantially reduces the bacterial load in the immune tissues of carp, particularly in the hepatopancreas and spleen [34]. Similarly, in the present study, the bacterial loads in major mucosal and systemic immune tissues (intestinal tract, hepatopancreas, gills, and kidneys) of juvenile *M. amblycephala* were also significantly lower in the polysaccharide-supplemented groups than in the control group. This indicated that dietary fucoidan and laminarin protected juvenile *M. amblycephala* by reducing the tissue bacterial load, thereby reducing the damage caused by bacterial infection.

### 4.4. Effect of Dietary Fucoidan and Laminarin on Tissue Structure Stability

Histological characteristics are reliable indicators of the physiological effects of pathogens on fish [35]. The hepatopancreas is a vital immune and digestive organ in fish, and its health substantially affects physiological functions. The hepatopancreas contains a wide array of immune cells and is considered the primary frontline immune tissue in fish [36]. Hepatocytes can enhance innate immunity against aggressive pathogens by synthesizing and releasing innate immune proteins [37]. In the present study, dietary fucoidan and laminarin reduced edema degeneration and necrosis of the hepatopancreas caused by bacterial infection and facilitated a more rapid recovery. Fucoidan and laminarin therefore maintained the health of the hepatopancreas, improving immune defense and reducing mortality of juvenile *M. amblycephala*.

### 4.5. Fucoidan and Laminarin Enhance Innate Immune and Antioxidant Enzyme Activities

ACP, AKP, and LZM are crucial innate immune enzymes in aquatic animals and play important roles in immune defense against pathogen invasion. Consequently, they are considered important indicators of non-specific immunity [38]. LZM can dissolve glycoproteins on the surface of bacteria, and ACP serves as an enzyme marker for lysosomes with bactericidal effects. Therefore, increased activities of ACP and LZM indicate enhanced anti-infection ability of the host [39,40,41]. Following infection with *A. hydrophila* in the present study, LZM activity in the 2% laminarin group remained relatively high at certain time points, both before and after infection. The ACP activity in the laminarin-supplemented groups was higher before infection and remained low at most time points post-infection. These results suggested that dietary laminarin supplementation maintained a balance between enhancing the antimicrobial capacity of the host and preventing excessive inflammatory responses. AKP is a key innate immune biomarker in teleost fish, and its elevated activity is generally considered an indicator of enhanced antimicrobial immune response during early bacterial infection [42]. In the present study, AKP activity in most laminarin- and fucoidan-supplemented groups was substantially higher than that in the control group following *A. hydrophila* infection, suggesting that fucoidan and laminarin supplementation improved the antimicrobial capacity of the host.

The antioxidant system can eliminate excess free radicals produced by the body as a result of pathogen infection. SOD, CAT, and GST are important antioxidant enzymes in fish. SOD can effectively eliminate harmful free radicals within the body and decompose them into non-toxic H_2_O and O_2_ under the action of CAT [43,44]. However, SOD activity is inhibited when the concentration of superoxide anion produced by the body exceeds the clearance capacity. The present study found that CAT activity in most polysaccharide-supplemented groups was higher than that in the control group, and SOD activity in the laminarin-supplemented groups was higher than that in the other groups, indicating that fucoidan and laminarin supplementation could enhance the antioxidant capacity of the host [45,46]. The fucoidan- and laminarin-supplemented groups showed higher GST activity than the control group at 0 and 6 hpi, and the levels subsequently stabilized, suggesting that the polysaccharide-supplemented groups regulated GST activity and exerted antioxidant effects in the early stages post-infection.

In summary, dietary fucoidan and laminarin enhanced the antioxidant capacity of juvenile *M. amblycephala* by regulating the activity of CAT, SOD, and GST, which may have improved the post-infection survival rate.

### 4.6. Effects of Dietary Fucoidan and Laminarin on Immune-Related Gene Expression

Fucoidan and laminarin may influence fish immune functions by activating or inhibiting specific signaling pathways, as evidenced by the alterations in the expression of immune-related genes within these pathways. Fucoidan boosts macrophage phagocytosis and lymphocyte activation by stimulating the relevant signaling pathways, resulting in increased interleukin production [47]. Fucoidan may alter the intestinal microbiota of zebrafish to an anti-inflammatory state by selectively inhibiting bacterial groups associated with pro-inflammatory responses, which is accompanied by the downregulation of the expression of related genes to achieve an immune effect [48]. Laminarin has been demonstrated to be an effective initiator of early signal transduction events, including the regulation of cytosolic [Ca^2+^] variations, H_2_O_2_ production, plasma membrane depolarization, and mitogen-activated protein kinase activation [49]. These signals subsequently induce the expression of defense-related genes that code for the production of disease-responsive proteins, including chitinase, glucanase, phenolic antimicrobial compounds, and compounds linked to cell wall reinforcement [49,50,51]. High-dose laminarin has been shown to promote the expression of pro-inflammatory cytokines in RTgutGC cells derived from the intestinal epithelium of rainbow trout [52].

As important anti-inflammatory cytokines in fish, *TGFβ*, *IL13*, and *IL10* can regulate the expression levels of pro-inflammatory cytokines such as *TNF*α and *IL1β*, thereby controlling the development of inflammation and mitigating the inflammatory response [53,54]. In the present study, the expression levels of pro-inflammatory cytokines and the transcript level of the acute-phase protein *SAA* in the hepatopancreas and kidneys initially increased and then gradually decreased upon infection in juvenile *M. amblycephala*. The polysaccharide-supplemented groups showed higher expression levels before 6 hpi but exhibited lower levels after 24 hpi. Upon infection, the expression patterns of anti-inflammatory cytokines were opposite to those of pro-inflammatory cytokines, which might assist in maintaining the host immune balance. These results suggest that dietary fucoidan and laminarin can initially enhance host immunity and subsequently prevent body damage from excessive inflammatory responses.

### 4.7. Immune-Related Protein Expression in Fucoidan- and Laminarin-Supplemented Fish

The present study investigated the protein expression levels in the hepatopancreatic and kidneys of juvenile *M. amblycephala* using Western blotting to assess the effects of dietary fucoidan and laminarin supplementation. TNFα, IL1β, and iNOS expression levels initially decreased and subsequently increased, with the majority of the polysaccharide-supplemented groups exhibiting lower expression than the control group, suggesting that dietary fucoidan and laminarin addition might prevent damage caused by excessive inflammatory responses by regulating the overexpression of pro-inflammatory cytokines and related inflammatory mediators.

To use fucoidan and laminarin as cost-effective fish feed additives, the focus should be on optimizing the sourcing of raw materials (e.g., using by-products from brown algae), simplifying the extraction and purification processes (e.g., using low-cost extraction methods and reducing the number of purification steps), and leveraging synergistic blends with other lower-cost additives. Scaling up production and encouraging industry collaboration will also help reduce costs. Thus, fucoidan and laminarin have potential as economically viable components of sustainable aquafeed, thereby cost-effectively enhancing fish health and productivity. It should be noted that the findings of this study are representative of juvenile *M. amblycephala* under the tested conditions, and longer-term trials are required to verify whether similar effects persist across later developmental stages.

## 5. Conclusions

The present study investigated the effects of dietary fucoidan and laminarin on the growth performance, mortality rate, tissue bacterial load, tissue structure stability, activities of innate immune and antioxidant enzymes, and expression patterns of immune-related genes and proteins in juvenile *M. amblycephala*. Dietary supplementation with 2% fucoidan significantly improved the growth performance and feed utilization of juvenile *M. amblycephala*, while 0.5% fucoidan and 2% laminarin only enhanced feed utilization. Additionally, both polysaccharides reduced the mortality rate caused by *A. hydrophila* infection. Moreover, dietary fucoidan and laminarin supplementation enhanced the anti-infection ability in juvenile *M. amblycephala* by reducing the tissue bacterial load, maintaining tissue structural integrity, modulating the activities of innate immune and antioxidant enzymes, and regulating immune-related gene and protein expression. Consequently, fucoidan and laminarin can be used as functional feed additives and immunostimulants in aquaculture.

## Figures and Tables

**Table 1:** Ingredients and nutrient composition of experimental diets.

Ingredient (% Dry Matter)	Group				
	Control	0.5% Fucoidan	2% Fucoidan	0.5% Laminarin	2% Laminarin
Fish meal	8	8	8	8	8
Soybean meal	20.7	20.7	20.7	20.7	20.7
Cottonseed meal	15	15	15	15	15
Rapeseed meal	18	18	18	18	18
Wheat middlings	30	29.5	28	29.5	28
Soybean oil	5	5	5	5	5
Ca(H_2_PO_4_)_2_	2	2	2	2	2
Seaweed polysaccharides	0	0.5	2	0.5	2
Choline	0.3	0.3	0.3	0.3	0.3
Vitamin premix ^(1)^	0.5	0.5	0.5	0.5	0.5
Mineral premix ^(2)^	0.5	0.5	0.5	0.5	0.5
Total	100	100	100	100	100
Nutrient composition (% dry matter)					
Moisture	6.32	6.30	6.38	6.29	6.39
Crude lipid	8.25	7.89	7.81	7.93	7.88
Crude protein	36.54	36.32	36.39	36.41	36.50
Ash	7.10	7.03	7.09	7.05	7.10
Energy (MJ/kg)	15.06	14.97	14.94	14.99	14.96

Note: (1) Vitamin premix per kilogram of feed: Vitamin E, 50 mg; Vitamin A, 5000 IU; Vitamin B1, 8 mg; Vitamin K, 5 mg; Vitamin B6, 8 mg; Vitamin D, 2000 IU; Vitamin B2, 10 mg; pantothenic acid, 30 mg; Vitamin B12, 0.03 mg; folic acid, 3 mg; niacin, 30 mg; inositol, 100 mg; biotin, 0.4 mg; and Vitamin C, 180 mg. (2) Mineral premix per kilogram of feed: magnesium, 300 mg; zinc, 150 mg; iron, 170 mg; cobalt, 0.25 mg; copper, 4 mg; manganese, 22 mg; and selenium, 0.4 mg.

**Table 2:** Primers used for quantitative reverse transcription polymerase chain reaction (qRT-PCR) in the present study.

Name	Sequence (5′-3′)
q*IL1β*-F	CGATAAGACCAGCACGACCTT
q*IL1β*-R	GTTTCCGTCTCTCAGCGTCA
q*IL6*-F	AAGACAACCGCACACTCGAT
q*IL6*-R	CTGGGTCTCTTCACGCCTTT
q*IL8*-F	TATTGTTGCTGTGGCATTTGTG
q*IL8*-R	TGGTTTCCTTCAGGGTGGC
q*IL10*-F	GTGTTTTCGGGTGCAAGTGG
q*IL10*-R	ATGAACGAGATCCTGCGCTT
q*IL13*-F	GCTGAGCAAGAACTGAAGACG
q*IL13*-R	TGGTTCCTTCATGGTGGTTGT
q*iNOS*-F	ATTCAAGGGCAGCTTCCAGG
q*iNOS*-R	CAGGGGCAAAGTTTAAGGGC
q*TNFα*-F	TGATGACGGCATTTACTTCG
q*TNFα*-R	CCTCCATAGGAATCAGAATAGC
q*TGFβ*-F	ACTGGACAAACAGAGAGGCG
q*TGFβ*-R	CAGGGGAGTTGCCGTTAGAG
q*C3*-F	ATCCTCAAGGCATCACACTCG
q*C3*-R	TTCAAAGCTCGGCAACACATA
q*SAA*-F	GTGCTGGTGCTGGGTTTGGT
q*SAA*-R	GCCTTCCTCATATCCTGGTAGGC
q*16S rRNA*-F	GGGAGTGCCTTCGGGAATCAGA
q*16S rRNA*-R	TCACCGCAACATTCTGATTTG
q*GAPDH*-F	TGCCGGCATCTCCCTCAA
q*GAPDH*-R	TCAGCAACACGGTGGCTGTAG
q*EF1α*-F	CTTCTCAGGCTGACTGTGC
q*EF1α*-R	CCGCTAGCATTACCCTCC
q*β-actin*-F	GCTCTTACAGGAAACGGGTC
q*β-actin*-R	GCAGCAGCTCTGTAGGTCAT

**Table 3:** Details of primary antibodies used for Western blotting.

Antibody	Host	Source	Dilution Ratio
TNFα	Rabbit	Self-made	1:500
IL1β	Rabbit	Self-made	1:500
iNOS	Rabbit	Self-made	1:500
GAPDH	Rabbit	GeneTex (Catalog No. GTX82899)	1:3000

Note: The antibodies directed against tumor necrosis factor alpha (TNFα, NCBI Protein Accession No.: ANA78340.1), interleukin-1 beta (IL1β, NCBI Protein Accession No.: QIQ08144.1), and inducible nitric oxide synthase (iNOS, NCBI Protein Accession No.: AIE77026.1) were prepared by our laboratory. The specificity of these recombinant proteins and their corresponding antibodies has been validated and is shown in Supplemental Figure S1.

**Table 4:** Effects of dietary supplementation with fucoidan and laminarin on growth and feed conversion ratios of juvenile *M. amblycephala*.

Item	Group				
	Control	0.5% Fucoidan	2% Fucoidan	0.5% Laminarin	2% Laminarin
Initial weight (g)	9.82 ± 1.65	9.66 ± 1.59	9.71 ± 1.19	10.76 ± 1.62	9.83 ± 1.33
Final weight (g)	32.66 ± 8.37	34.16 ± 7.96	40.56 ± 7.96	32.54 ± 6.24	37.50 ± 5.49
Weight gain rate (%)	234.26 ± 68.73 ^bc^	255.28 ± 65.99 ^abc^	319.72 ± 75.63 ^a^	206.15 ± 57.84 ^c^	287.11 ± 72.85 ^ab^
Feed conversion ratio	1.59 ± 0.02 ^a^	1.49 ± 0.03 ^b^	1.42 ± 0.03 ^c^	1.57 ± 0.04 ^a^	1.38 ± 0.04 ^c^
Survival rate (%)	96.67 ± 3.33	98.89 ± 1.92	98.89 ± 1.92	97.78 ± 1.92	98.89 ± 1.92

Note: Data are mean values ± SE. Different letters indicate statistically significant differences among different groups (*p* < 0.05).

## Data Availability

The original data presented in this study are openly available in Figshare at doi: 10.6084/m9.figshare.31925562.

## Supplementary Materials

The following supporting information can be downloaded at https://www.mdpi.com/article/10.3390/ani16131989/s1: Supplemental Figure S1: Detection of purified recombinant TNFα, IL-1β, and iNOS proteins and the prepared antibodies; Supplemental Figure S2: Effects of fucoidan and laminarin on the expression levels of immune-related proteins in juvenile *M. amblycephala*. (A) Western blotting analysis of TNFα, IL1β, and iNOS protein levels. The relative protein levels of TNFα, IL1β, and iNOS in the hepatopancreas (B) and kidneys (C) were determined using the gray values of Western blotting. Asterisks indicate statistically significant differences compared to the control group (*p* < 0.05). Supplemental Figure S3: Original Western blotting images of proteins in the hepatopancreas. (A–D) were the target proteins of TNFα, IL-1β, iNOS, and GAPDH, respectively. ①–④ indicated different time points, ①: 0 hpi, ②: 6 hpi, ③: 24 hpi, ④: 72 hpi. M and 1–5 represented different groups, M: Marker, 1: Control, 2: 0.5% Fucoidan, 3: 2% Fucoidan, 4: 0.5% Laminarin, 5: 2% Laminarin. Arrows: target bands. Supplemental Figure S4: Original Western blotting images of proteins in the kidneys. (A–D) were the target proteins of TNFα, IL-1β, iNOS, and GAPDH, respectively. ①–④ indicated different time points, ①: 0 hpi, ②: 6 hpi, ③: 24 hpi, ④: 72 hpi. M and 1–5 represented different groups, M: Marker, 1: Control, 2: 0.5% Fucoidan, 3: 2% Fucoidan, 4: 0.5% Laminarin, 5: 2% Laminarin. Arrows: target bands.

## Abbreviations

The following abbreviations are used in this manuscript:

*C3*	*Complement component 3*
CAT	Catalase
Ct	Threshold cycle
ACP	Acid phosphatase
AKP	Alkaline phosphatase
*GAPDH*	*Glyceraldehyde-3-phosphate dehydrogenase*
GST	Glutathione S-transferase
H&E	Hematoxylin and eosin
hpi	Hours post-infection
HRP	Horseradish peroxidase
*IL1β*	*Interleukin-1 beta*
*IL6*	*Interleukin-6*
*IL8*	*Interleukin-8*
*IL10*	*Interleukin-10*
*iNOS*	*Inducible nitric oxide synthase*
LD	Linear dichroism
LZM	Lysozyme
PVDF	Polyvinylidene difluoride
qRT-PCR	Quantitative reverse transcription polymerase chain reaction
*SAA*	*Serum amyloid A*
SE	Standard error
SOD	Superoxide dismutase
*TGFβ*	*Transforming growth factor beta*
*TNFα*	*Tumor necrosis factor alpha*
