# Supplementary material for: Immunoprotective Effects of Dietary Fucoidan and Laminarin on Juvenile Blunt Snout Bream (Megalobrama amblycephala)
Source: Animals (Basel). 2026 Jun 27;16(13):1989. doi: 10.3390/ani16131989 (PMC13360614; doi:10.3390/ani16131989)
Supplement: Supplementary file 1 [file animals-16-01989-s001.zip › animals-4265649-supplementary.pdf]

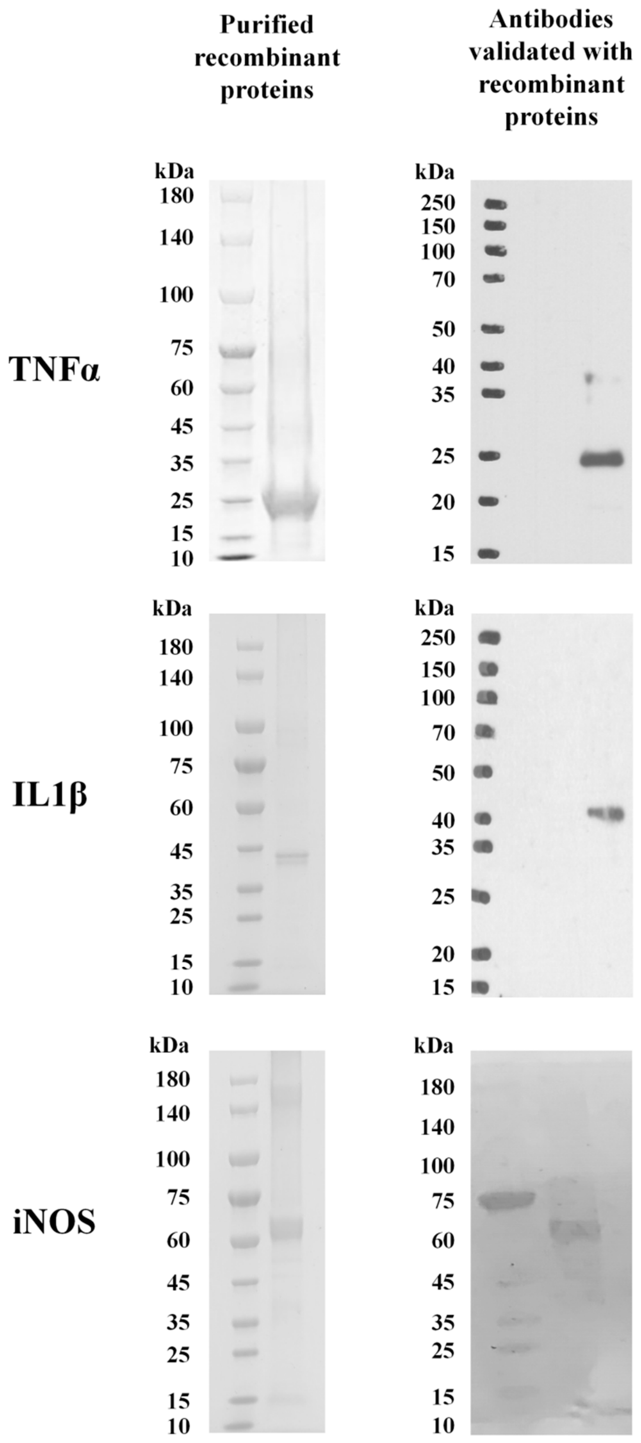

Supplemental Figure S1. Detection of purified recombinant TNFα, IL-1β, and iNOS proteins and the prepared antibodies.

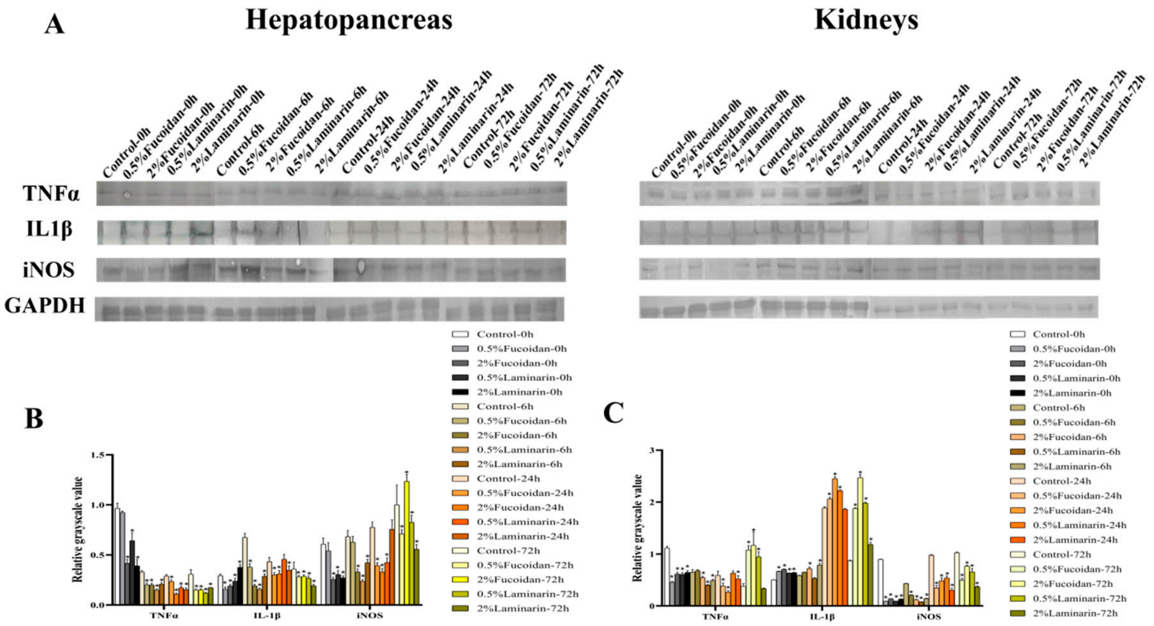

Supplemental Figure S2. Effects of fucoidan and laminarin on the expression levels of immune-related proteins in juvenile *M. amblycephala*.

(A) Western blotting analysis of TNFα, IL1β, and iNOS protein levels. The relative protein levels of TNFα, IL1β, and iNOS in the hepatopancreas (B) and kidneys (C) were determined using the gray values of Western blotting. Asterisks indicate statistically significant differences compared to the control group ($P < 0.05$).

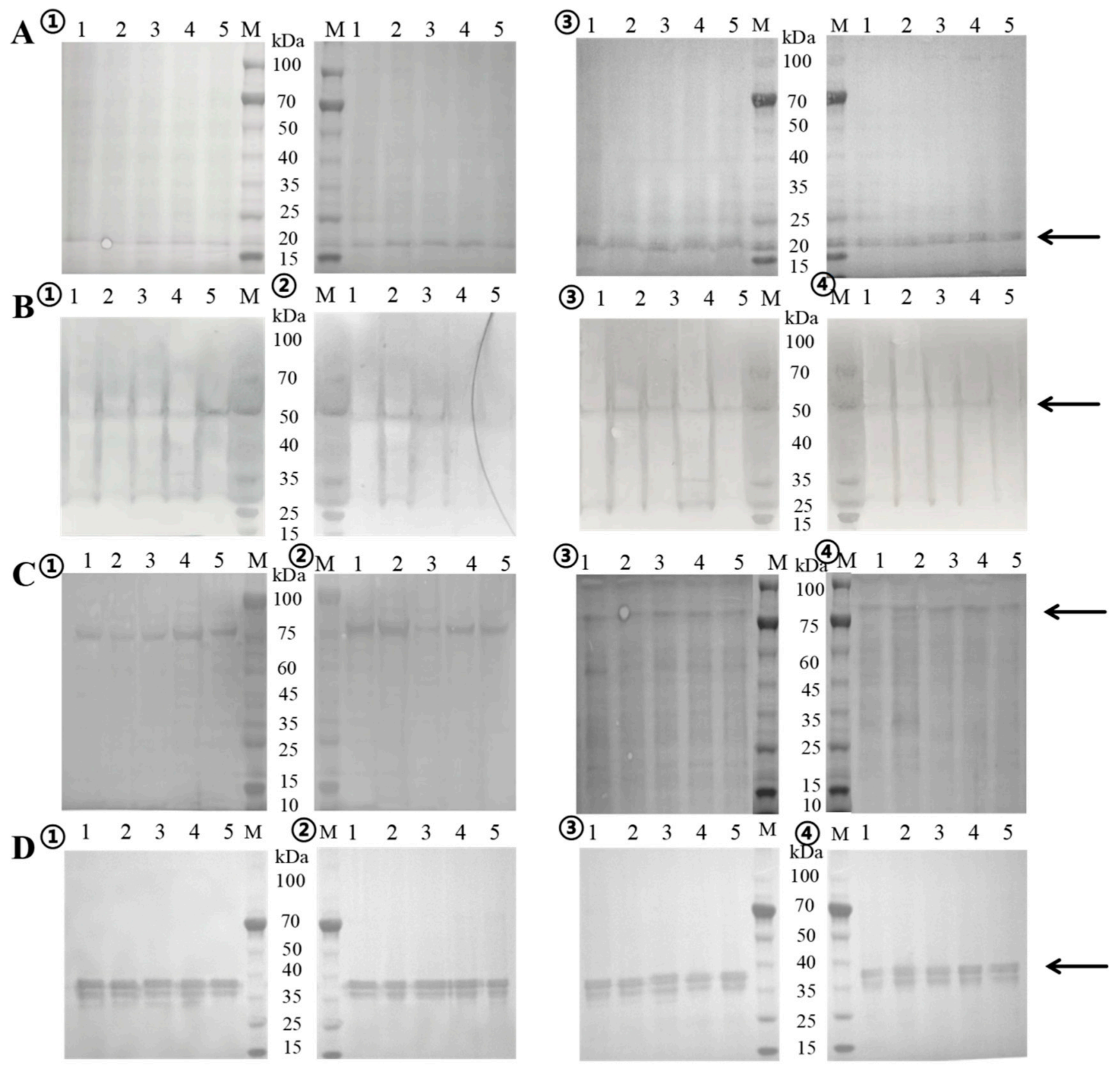

Supplemental Figure S3. Original western blotting images of proteins in the hepatopancreas.

(A)-(D) were the target proteins of TNFα, IL-1β, iNOS, and GAPDH, respectively.

①-④ indicated different time points, ①: 0 hpi, ②: 6 hpi, ③: 24 hpi, ④: 72 hpi.

M and 1-5 represented different groups, M: Marker, 1: Control, 2: 0.5% Fucoidan, 3: 2% Fucoidan, 4: 0.5% Laminarin, 5: 2% Laminarin. Arrows: target bands.

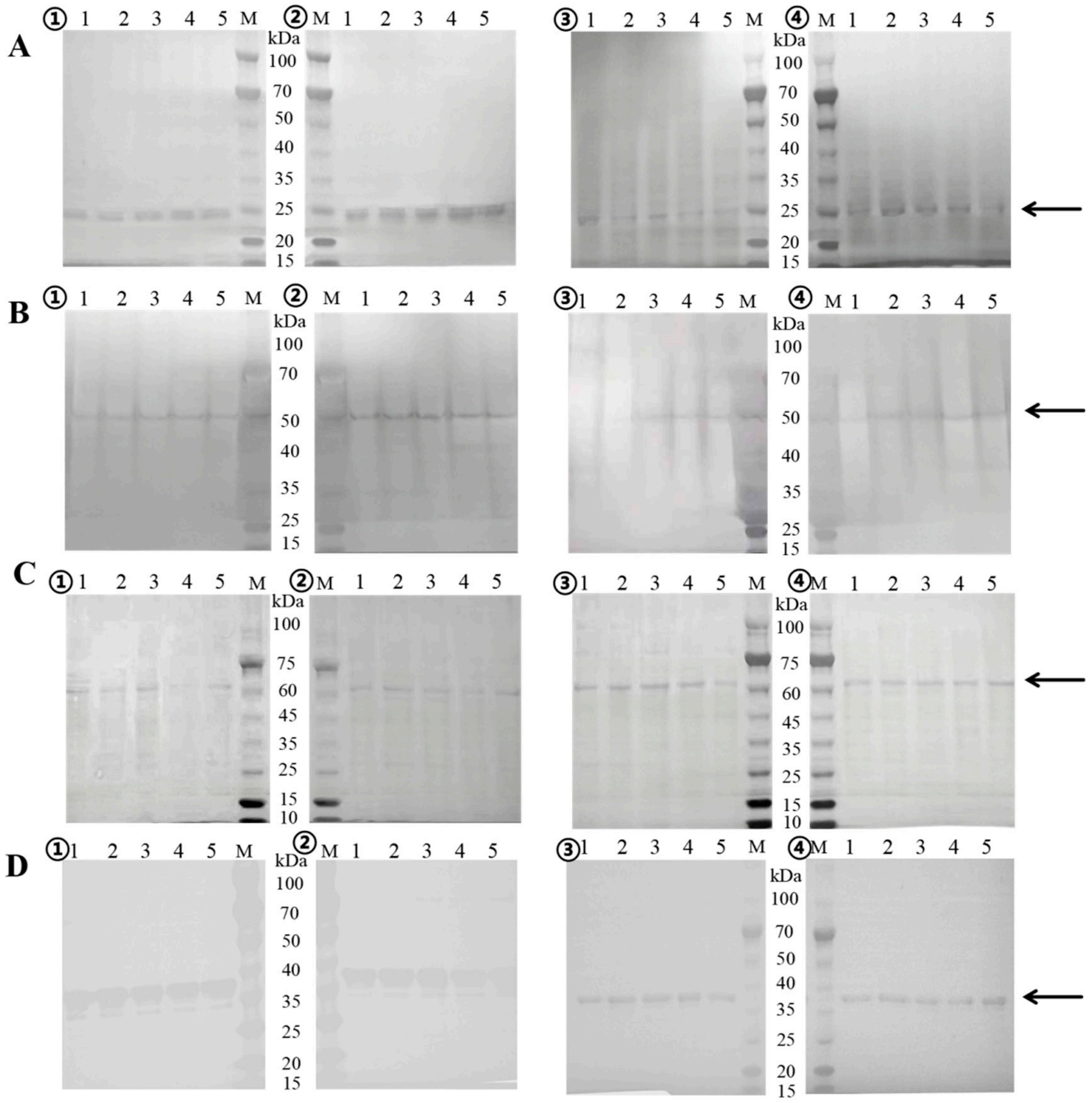

Supplemental Figure S4. Original western blotting images of proteins in the kidneys. (A)-(D) were the target proteins of TNFα, IL-1β, iNOS, and GAPDH, respectively. ①-④ indicated different time points, ①: 0 hpi, ②: 6 hpi, ③: 24 hpi, ④: 72 hpi. M and 1-5 represented different groups, M: Marker, 1: Control, 2: 0.5% Fucoidan, 3: 2% Fucoidan, 4: 0.5% Laminarin, 5: 2% Laminarin. Arrows: target bands.
